# Novel Biomarker Genes for Prognosis of Survival and Treatment of Glioma

**DOI:** 10.3389/fonc.2021.667884

**Published:** 2021-12-15

**Authors:** Xiaopeng Zhu, Sian Pan, Rui Li, Zebo Chen, Xingyun Xie, Deqing Han, Shengqing Lv, Yongkai Huang

**Affiliations:** ^1^ Department of Neurosurgery, Zhuzhou Central Hospital, Zhuzhou, China; ^2^ Department of Rehabilitation Medicine, Zhuzhou Central Hospital, Zhuzhou, China; ^3^ Department of Operating Theatre, Zhuzhou Central Hospital, Zhuzhou, China; ^4^ Department of Neurosurgery, Xinqiao Hospital, Third Military Medical University, Chongqing, China

**Keywords:** glioblastoma, prognosis, GEO, radiotherapy, chemotherapy, immunotherapy, TMB

## Abstract

Glioblastoma multiforme (GBM) is the most aggressive malignant primary central nervous system tumor. Although surgery, radiotherapy, and chemotherapy treatments are available, the 5-year survival rate of GBM is only 5.8%. Therefore, it is imperative to find novel biomarker for the prognosis and treatment of GBM. In this study, a total of 141 differentially expressed genes (DEGs) in GBM were identified by analyzing the GSE12657, GSE90886, and GSE90598 datasets. After reducing the data dimensionality, Kaplan-Meier survival analysis indicated that expression of PTPRN and RIM-BP2 were downregulated in GBM tissues when compared with that of normal tissues and that the expression of these genes was a good prognostic biomarker for GBM (p<0.05). Then, the GSE46531 dataset and the Genomics of Drug Sensitivity in Cancer (GDSC) database were used to examine the relationship between sensitivity radiotherapy (RT) and chemotherapy for GBM and expression of PTPRN and RIM-BP2. The expression of PTPRN was significantly high in RT-resistant patients (p<0.05) but it was not related to temozolomide (TMZ) resistance. The expression level of RIM-BP2 was not associated with RT or TMZ treatment. Among the chemotherapeutic drugs, cisplatin and erlotinib had a significantly good treatment effect for glioma with expression of PTPRN or RIM-BP2 and in lower-grade glioma (LGG) with IDH mutation. (p < 0.05). The tumor mutational burden (TMB) score in the low PTPRN expression group was significantly higher than that in the high PTPRN expression group (p=0.013), with a large degree of tumor immune cell infiltration. In conclusion, these findings contributed to the discovery process of potential biomarkers and therapeutic targets for glioma patients.

## Introduction

An estimated 86,010 new cases of primary brain and other central nervous system (CNS) tumors were diagnosed in the US in 2019 (1). Glioblastoma multiforme (GBM) is the most common and aggressive primary CNS tumor (2). Despite the availability of several treatment options, including surgery, radiotherapy, and chemotherapy, the median overall survival (OS) of GBM remains approximately 15 months, and the 5-year survival rate is 5.8% (3). In 2016, the updated World Health Organization (WHO) classification was the first to integrate molecular parameters with histology to define many tumor entities, including GBM (4), thus formulating a new concept for how GBM diagnoses should be structured in the molecular era. Although IDH1/2 mutations, MGMT promoter methylation, and 1p/19q loss have been recognized as appropriate diagnostic and prognostic markers (5, 6), patients with GBM still have poor outcomes, with one of the worst 5-year OS rates among all human cancers (7). Therefore, it is vital to develop appropriate and effective novel molecular signatures to improve survival and treatment response prediction for patients with GBM. With the development of next-generation sequencing (NGS) technologies, a large amount of data on differentially expressed genes (DEGs), non-coding RNAs, and protein modifications have been identified and stored in public databases. Gene Expression Omnibus (GEO, https://www.ncbi. nlm.nih.gov/geo/), The Cancer Genome Atlas (TCGA, https://portal.gdc.cancer.gov), and Chinese Glioma Genome Atlas (CGGA, http://www.cgga.org.cn) provide us with the opportunity and resources to explore, integrate, and reanalyze the existing data for new GBM biomarker discovery.

Although genomic analysis of cancers is at the forefront of drug and molecular pathogenesis discovery (8), much of the research has focused on biomarkers related to GBM prognosis. Only a few studies have explored potential therapeutic options related to novel molecular signatures.

In this study, bioinformatics methods were used, and we found two potential markers, PTPRN and RIM-BP2, associated with OS in patients with GBM. Furthermore, our goal was to provide information for designing radiotherapy and chemotherapy regimens by monitoring these biomarkers.

## Materials and Methods

### Data Source

The Series Matrix Files for gene expression microarray datasets were downloaded from the National Center of Biotechnology Information (NCBI) Gene Expression Omnibus(GEO). GBM tumor samples smaller than 6 and without normal or adjacent tumor tissue in GEO data were considered inappropriate samples in this study. In addition, to rule out interference, samples that had undergone chemotherapy or radiotherapy were also excluded.Among them, 3 independent GEO datasets, GSE12657, GSE90886, and GSE90598, including 7 samples of GBM and 5 samples of normal brain tissue, 9 samples of GBM and 9 samples of normal brain tissue, and 16 samples of GBM and 7 samples of normal brain tissue, respectively, were included. The dataset was based on the GPL8300, GPL15207, and GPL17692 platforms of the Affymetrix Human Genome U133 Plus 2.0 Array (Affymetrix, Santa Clara, CA, United States). Then, gene profiles were standard normalized by spatially variant apodization (SVA) within and among samples. To analyze the sensitivity of radiotherapy based on hub genes, the Gene expression microarray dataset GSE46531, which is only qualified and relevant data, was extracted for subsequent analysis. Glioma stem cell(GSC)culture lines were established from fresh GBM tumors. Treatment-resistant clones, including sensitive clones (n=6), RT-resistant clones (n = 3) and RT+TMZ-resistant clones (n = 3), obtained by irradiating the cultured cells with a certain dose of radiation and adding TMZ to the cell culture, were used for microarray analysis to explore different molecules involved in response therapy (9). The GBM RNA sequencing data (RNA-seq) were downloaded from the TCGA database (https://portal.gdc.cancer.gov). A total of 174 RNA-seq datasets were extracted for subsequent validation.

### Gene Ontology (GO) and Kyoto Encyclopedia of Genes and Genomes (KEGG) Annotation

GO and KEGG enrichment analyses were performed on the survival-related genes. The Metascape database (www.metascape.org) was used to annotate and visualize GO terms and KEGG pathways. Min overlap≥3 and P < 0.01 were set as threshold values.

### Identification of Optimal Diagnostic Gene Biomarkers

The LASSO algorithm was applied with the glmnet package (https://cran.r-project.org/web/packages/glmnet/) (10). The Boruta algorithm (https://cran.r-project.org/web/packages/Boruta/) employs a wrapper approach built around a random forest classifier (11). The DEGs between GBM and normal controls were retained for feature selection, and biomarker genes for GBM were identified with the above algorithms. The optimal biomarker genes for GBM were then identified by overlapping the biomarkers derived from these two algorithms. Based on these optimal gene biomarkers, the Boruta package was used further to evaluate the diagnostic value of these biomarkers in GBM.

### Tumor-Infiltrating Immune Cell Analysis

The CIBERSORT package was used to explore the differences in immune cell subtypes including B cells, T cells, natural killer (NK) cells, macrophages, and dendritic cells (DCs), based on the expression data (12). Samples with P < 0.05 in CIBERSORT analysis results were used for further analysis. Spearman analysis was used to compare differences in immune cell subtypes in the high hub-gene and low hub-gene groups.

### Gene Set Variation Analysis (GSVA)

The gene set variation analysis (GSVA) method is nonparametric and unsupervised and bypasses the conventional approach of explicitly modeling phenotypes within the enrichment scoring algorithm (13). GSVA calculates samples gene set enrichment scores as a function of the genes inside and outside the gene set. Furthermore, it estimates the variation in gene set enrichment over the samples independently of any class label. In this study, gene sets were obtained from the Molecular Signatures Database v7.0. GSVA was used to compute single-sample enrichment scores to describe the potential changes in biological function.

### Drug Sensitivity Analysis

The largest publicly available pharmacogenomics database, Genomics of Drug Sensitivity in Cancer (GDSC, https://www.cancerrxgene.org/) has characterised 1000 human cancer cell lines and screened them 100s of compounds, was used to obtain drug IC50 values and predict the chemotherapeutic response of each sample (14). The prediction procedure was performed by the R software package “prophetic”. Tenfold cross-validation was used to assess the prediction accuracy based on the GDSC training set (15).

### Tumor Mutational Burden Analysis

The TMB was defined as the number of somatic, coding, base substitution, and indel mutations identified by next-generation sequencing (NGS). Mutations obtained from SNPs in GBM samples were downloaded from the database using VarScan2 and SAMtools (16). To estimate the TMB of the training set, we counted all coding somatic base substitutions and indels in the targeted regions, including **“**stop/start-loss/frameshift/missense/inframe**”** alterations.

### GeneMANIA Analysis

GeneMANIA (http://genemania.org) is a flexible, user-friendly website for generating hypotheses about gene functions, analyzing gene lists, and prioritizing genes for functional assays (17).Given a query gene list, GeneMANIA finds functionally similar genes using a wealth of genomics and proteomics data. In this mode, it weights each functional genomic dataset according to its predictive value for the query. In this study, GeneMANIA was used to visualize the molecular network analyses to explore possible hub genes and their mechanisms in GBM.

### Statistical Analysis

All P-values were two-sided, and values lower than 0.05 were considered significant. Statistical significance is indicated in the figures as follows: * P <0.05, * * P < 0.01. R Studio (version 3.6) and corresponding packages was used for all statistical analyses. The glmnet R package was used for LASSO analysis. Survival curves plotted by the Kaplan–Meier method were compared to the log-rank test. The mutation were analyzed by cBioportal package. The CIBERSORT package was used to explore the differences in immune cell subtypes. The prophetic package was performed to predict the chemotherapeutic response.

## Results

### Construction of a Prognostic Classifier Based on DEGs in GBM

**Figure 1:**
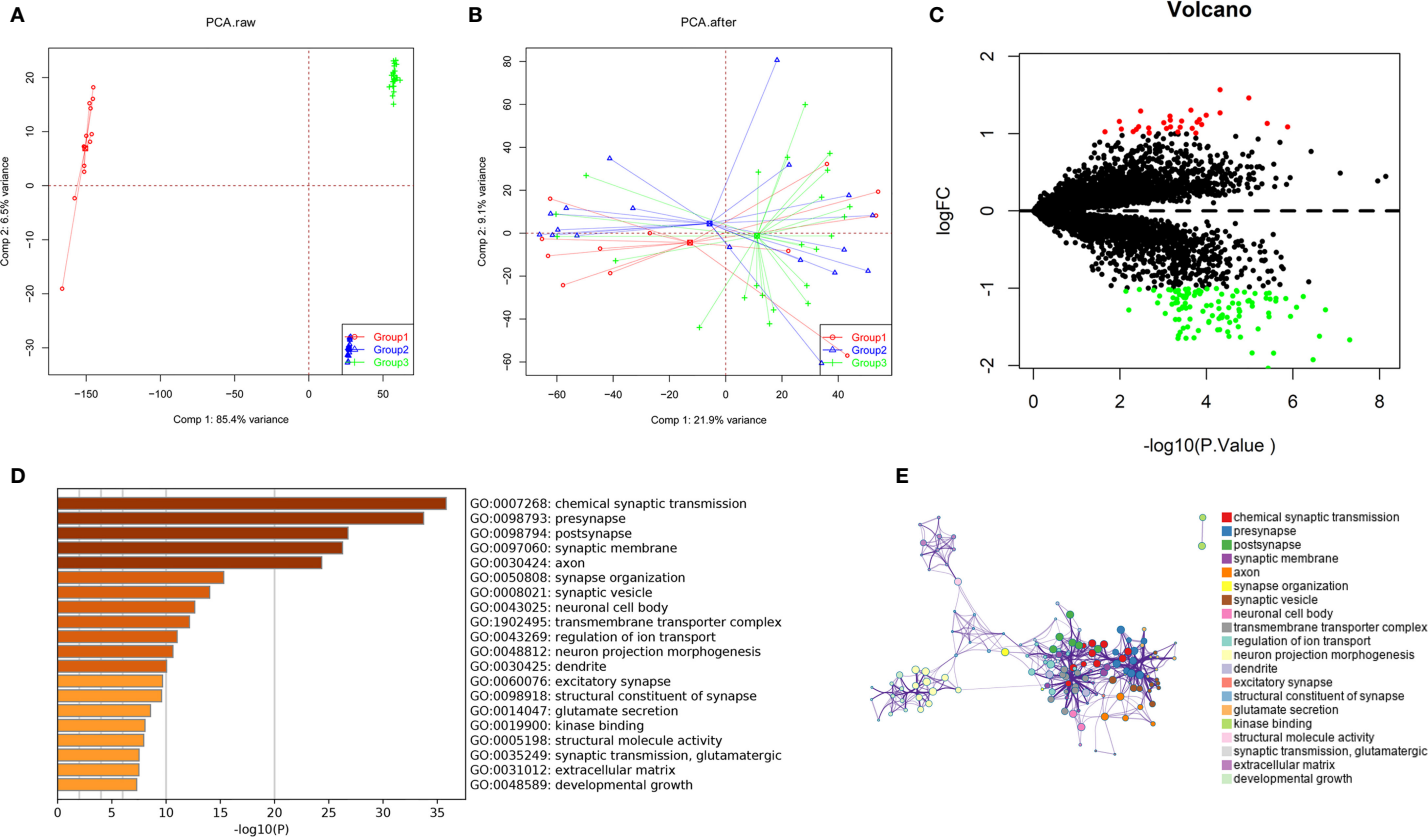
Identification of DEGs among GBM datasets from TCGA and GEO. **(A, B)** The SVA algorithm was used to normalize the datasets, and the PCA plot shows the batch effect. **(C)** Volcano plots of DEGs created by using the limma package. **(D, E)** GO and KEGG pathway enrichment analysis performed by the Metascape database.

GBM gene expression microarray data (GSE12657, GSE90886, and GSE90598) with a total of 53 samples (32 GBM and 21 control) were downloaded from the NCBI GEO. The spatial variant apodization (SVA) algorithm was used to normalize the datasets, and the principal component analysis (PCA) plot shows the batch effect before and after normalization (**Figures 1A, B**). The package limma was used to perform the data analysis. Fold change > 1 and p < 0.05 were set as the cutoffs to screen for DEGs. Compared with normal brain tissues, the limma package identified 141 DEGs (**Supplementary Table 1**) in GBM, of which 30 were upregulated and 111 were downregulated (**Figure 1C**). GO and KEGG pathway enrichment analyses suggested that these genes mainly participated in the following pathways: chemical synaptic transmission, presynapse, postsynapse, synaptic membrane, and axon (**Figures 1D, E**). Biological processes of DEGs were mainly associated with the chemical synaptic transmission that affects the neuronal activity and neurotransmitters to participate in the onset and progression of GBM (18, 19). Some synapse-related genes, such as RIM-BP2 and CACNG3, have been less studied in tumors.

**Figure 2:**
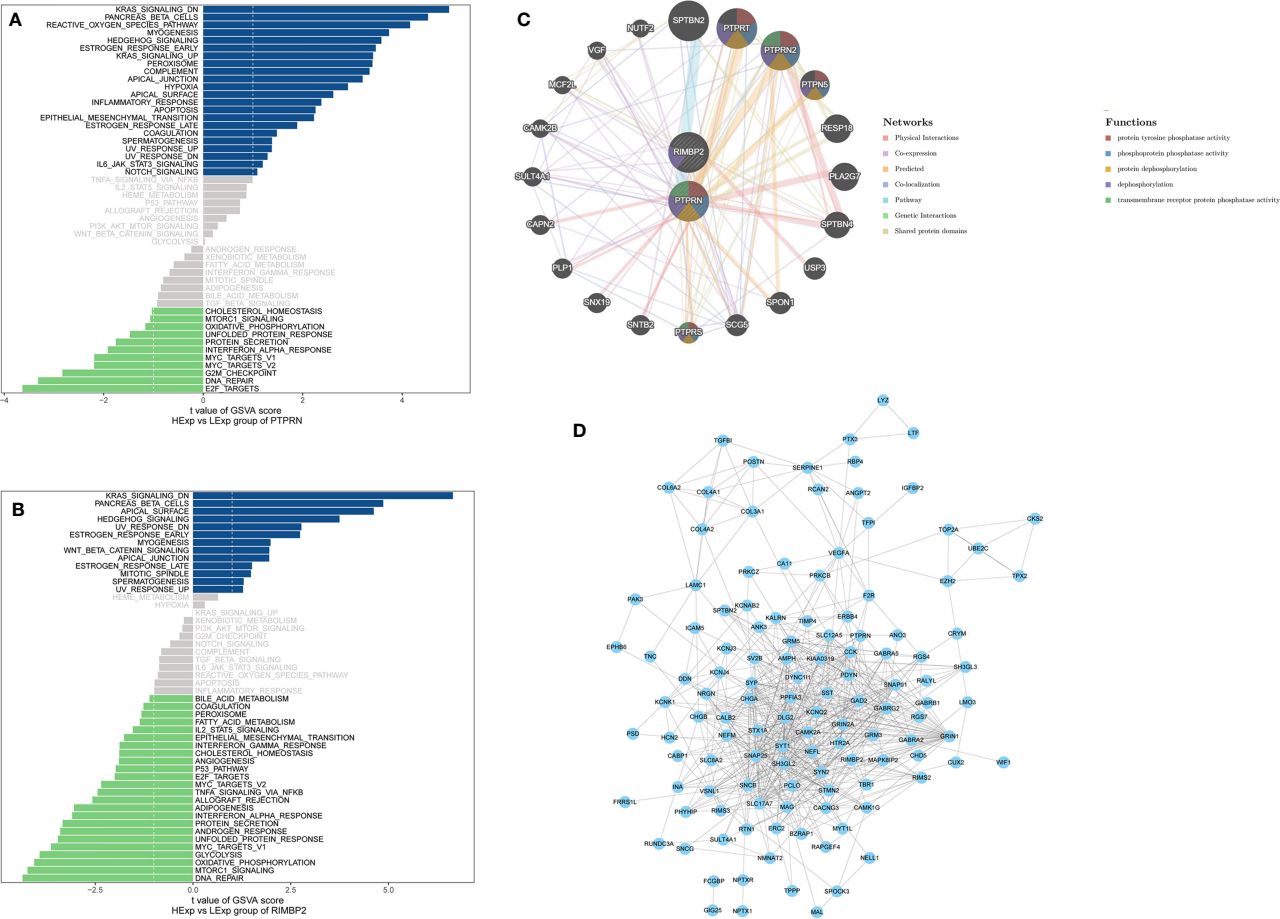
The pathway and coexpression networks of different levels of PTPRN and RIM-BP2 expression. **(A, B)** The GSVA database shows the pathways associated with different expression levels of PTPRN and RIM-BP2. **(C)** The coexpression network of the two-gene signature. **(D)** Visualization of the coexpression network of the DEGs was generated using Cytoscape. Based on weights, not all genes corresponding to each module were represented.

The results of the GSVA database analysis showed that differential expression of PTPRN and RIM-BP2 was involved in DNA repair and the APICAL_JUNCTION and APICAL_SURFACE ESTROGEN_RESPONSE_EARLY pathways (**Figures 2A, B**). Metascape was used to construct protein-protein interaction (PPI) networks (**Figure 2C**). The coexpression network of the DEGs is shown in **Figure 2D**.

**Figure 3:**
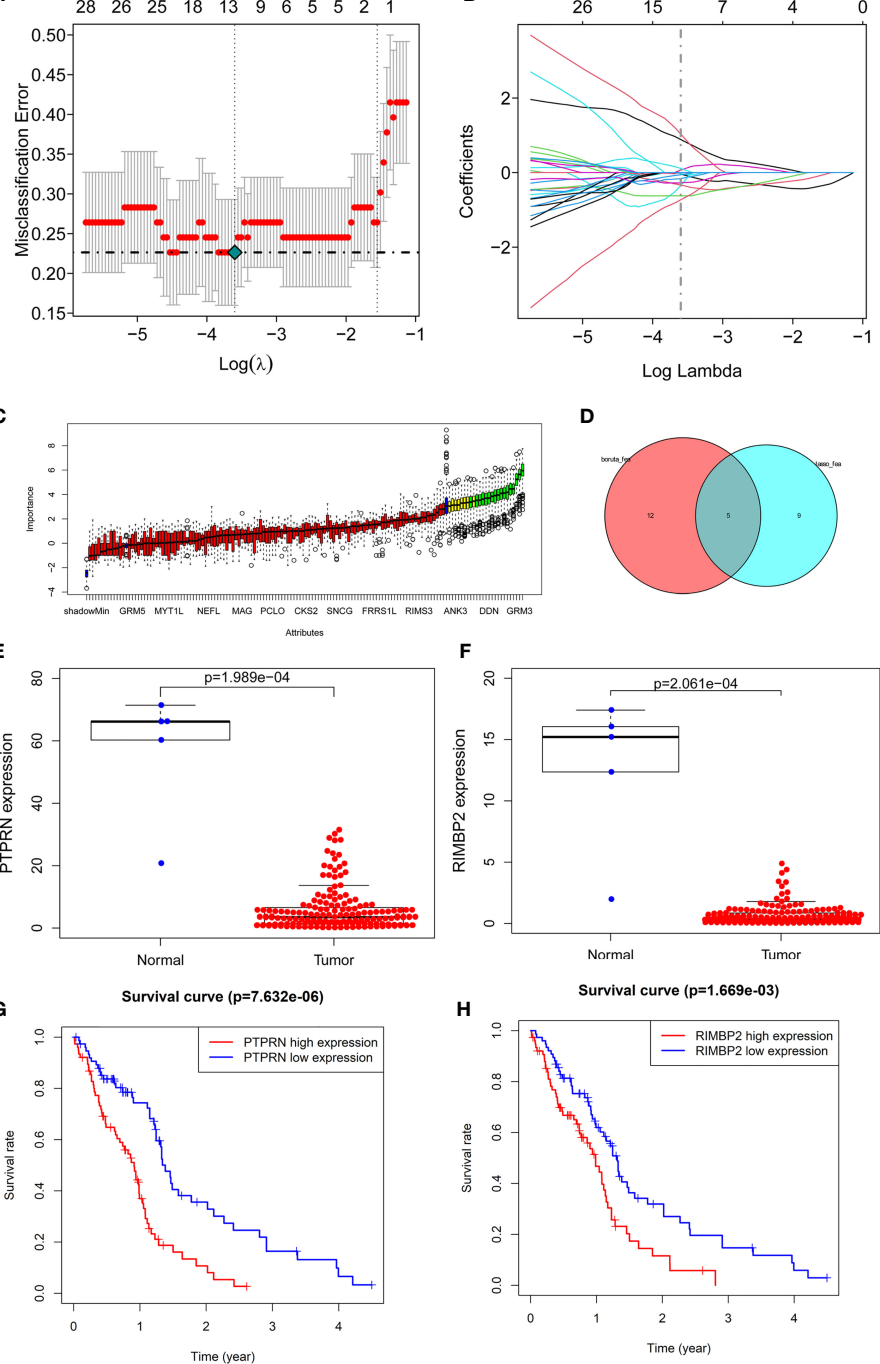
Identification of optimal survival biomarker genes **(A, B).** Determination of the number of factors by LASSO analysis. **(C)** Determination of the number of elements by the Boruta algorithm. **(D)** The Venn diagram of DEGs among the LASSO analysis and Boruta algorithm defined 5 hub genes. Among them, 2 genes (PTPRN and RIM-BP2) were associated with survival. **(E, F)** Expression of PTPRN and RIM-BP2 in normal and GBM tissues. **(G, H)** Kaplan–Meier analysis using the median risk score cutoff to divide patients into low gene expression and high gene expression groups.

**Figure 4:**
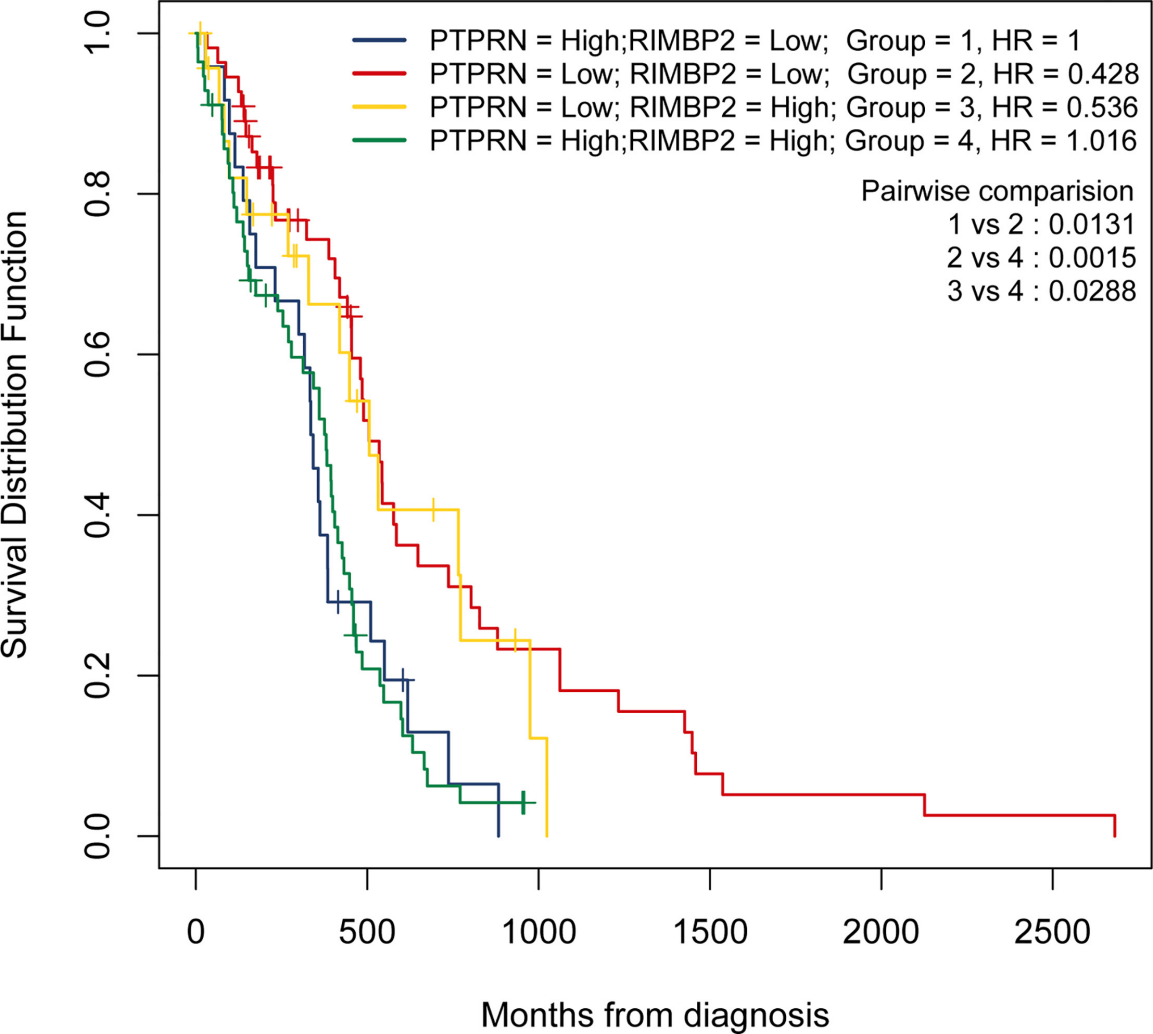
PTPRN and RIM-BP2 predict the prognosis of GBM. Kaplan-Meier curves of overall survival for GBM patients with different PTPRN and RIM-BP2 (combined) expression levels.

Next, we identified 14 DEGs as GBM survival-related genes to be included in the classifier using the LASSO analysis (**Figures 3A, B**). Boruta algorithm analysis identified 17 DEGs as survival-related genes (**Figure 3C**). Then, we obtained five DEGs, including SLC8A2, PTPRN, F2R, RIM-BP2, and IFI44, by overlapping the two analyses (**Figure 3D**); of these, PTPRN and RIM-BP2 were highly expressed in GBM (p<0.05) (**Figures 3E, F**). Kaplan-Meier survival curves from the TCGA database were used to explore the potential roles of individual DEGs in GBM OS. Among the five genes, high expression of PTPRN (p=7.632e-06) or RIM-BP2 (p=1.669e-03) significantly predicted poor overall survival (**Figures 3G, H**). Combined analysis of *PTPRN* and *RIM-BP2* showed no significant advantage for the prediction of GBM prognosis compared with either gene individual analysis. In addition, PTPRN was found to play the dominant role in prognosis prediction in the combined analysis of the two genes (**Figure 4**).

### Expression of PTPRN and RIM-BP2 in Response to Radiation Treatment (RT) and Drug Therapy in GBM

**Figure 5:**
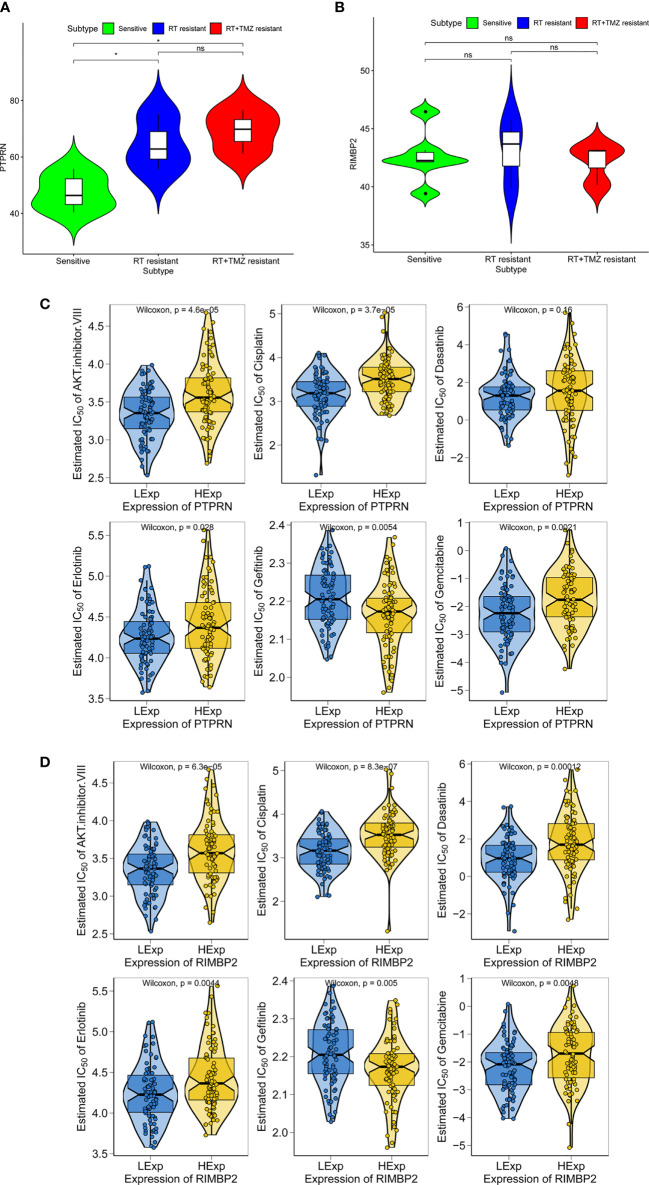
Expression of PTPRN and RIM-BP2 in response to radiation treatment (RT) and anticancer drugs in GBM. **(A, B)** Expression of PTPRN and RIM-BP2 insensitive, RT-resistant and RT+TMZ-resistant groups. **(C, D)** GBM sensitivity to standard chemotherapy drugs with respect to PTPRN and RIM-BP2 expression. *p < 0.05; ns, no significant.

To explore the relationship between expression of PTPRN and RIM-BP2 and the sensitivity to RT or TMZ, we first wanted to know whether the PTPRN and RIM-BP2 genes were differentially expressed in RT-resistant and RT+TMZ-resistant patients than RT-sensitive and RT+TMZ- sensitive patients. Six sensitive groups (three patients, two clones per patient), three RT-resistant groups, and three RT+TMZ-resistant groups were used for the analysis. The results showed that expression of PTPRN was significantly higher in the RT-resistant patient group and RT+TMZ-resistant group than in the sensitive groups (p<0.05). There were no differences in the expression of PTPRN between the RT-resistant group and the RT+TMZ-resistant group (**Figure 5A**). Moreover, the difference in the expression of RIM-BP2 was also not significant among the three groups (**Figure 5B**). These results suggested that the patients with higher PTPRN expression were more resistant to RT. TMZ treatment did not change the resistance to RT, suggesting that PTPRN expression was not associated with sensitivity to TMZ.

Chemotherapy is a common treatment for GBM. We further analyzed the sensitivity of PTPRN and RIM-BP2 to chemotherapy drugs, including AKT inhibitor VIII, cisplatin, erlotinib, gefitinib, and gemcitabine. The prediction model on the GDSC was used. 10-fold cross-validation for TCGA GBM cohort resulted in satisfactory prediction. The results showed that there were significant differences in PTPRN and RIM-BP2 expression in response to several drugs, suggesting that both PTPRN and RIM-BP2 were sensitive to common chemotherapy drugs (p < 0.05). According to the predictive model of chemotherapy drugs in TCGA dataset, the order of sensitivity responses PTPRN to chemotherapy drugs was AKT inhibitor VIII > cisplatin > erlotinib > gefitinib > gemcitabine. In contrast, the order of sensitivity responses of RIM-BP2 to chemotherapy drugs was AKT inhibitor VIII > cisplatin > dasatinib > erlotinib > gefitinib > gemcitabine (**Figures 5C, D**).

### Expression of PTPRN and RIM-BP2 in Response to Anticancer Drugs in LGG With IDH Mutation

LGG accounts for approximately 20% of primary malignant tumors of the CNS and occurs most commonly in young adults. According to the RTOG 9802 standard, LGG is clinically divided into a low-risk group and a high-risk group according to patient age, the occurrence of subtotal resection, and histology findings (20). RT is necessary to treat high-risk LGG, and the treatment appears to be effective in patients with IDH mutations. For patients with high-risk LGG, National Comprehensive Cancer Network (NCCN) guidelines recommend postoperative RT + procarbazine, lomustine, and vincristine (PCV) chemotherapy or RT + adjuvant TMZ chemotherapy or RT + synchronous adjuvant TMZ chemotherapy (20, 21). The appropriate postoperative treatment for patients with high-risk LGG remains under debate.

**Figure 6:**
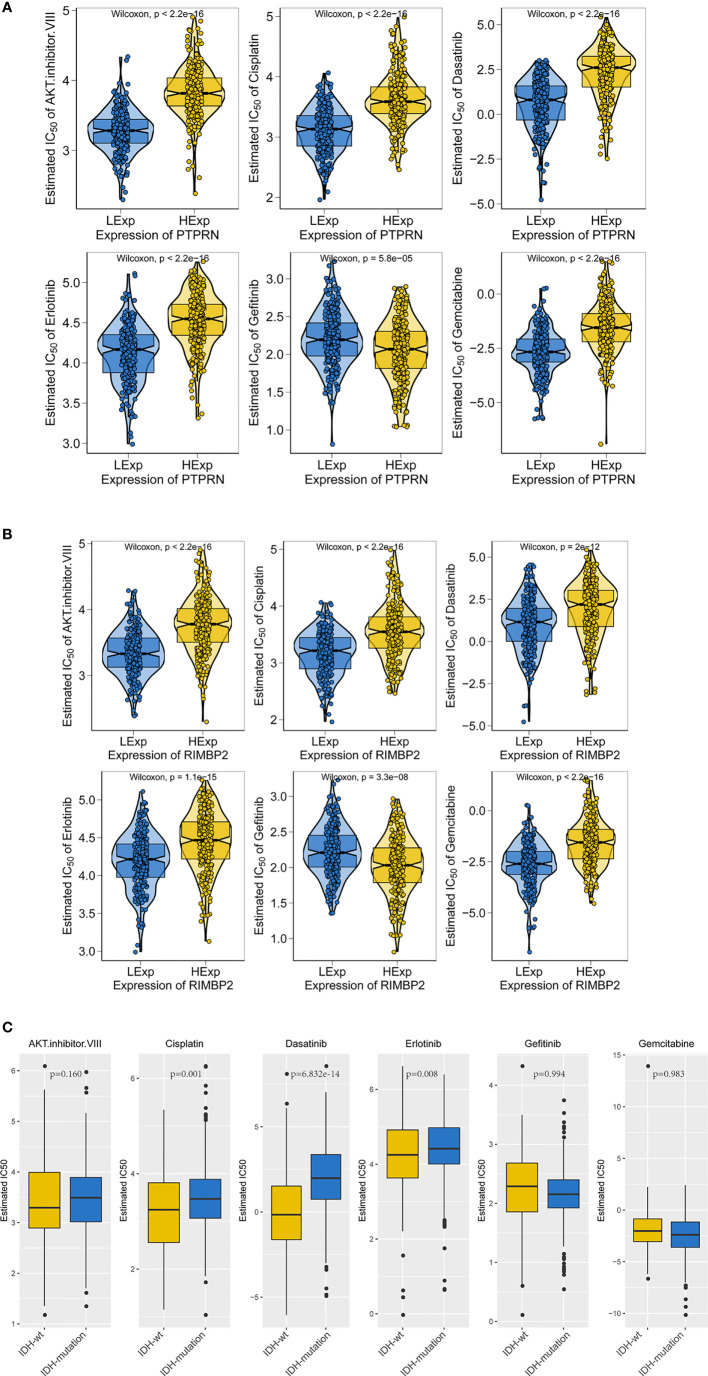
The gene expression and IDH mutation response to anticancer drugs in LGG. **(A, B)** Sensitivity to standard chemotherapy drugs relative to PTPRN and RIM-BP2 in LGG. **(C)** Sensitivity to traditional chemotherapy drugs comparable to IDH mutation in LGG.

Therefore, we evaluated the relationship between PTPRN and RIM-BP2 expression and chemotherapy in LGG, and the order of sensitivity in cases with high PTPRN and RIM-BP2 was as follows: AKT inhibitor VIII > cisplatin > dasatinib > erlotinib > gefitinib > gemcitabine (p < 0.05) (**Figures 6A, B**).

IDH mutations are common in LGG; thus, we analyzed the sensitivity of LGG tumors with IDH mutations to chemotherapy drugs. We found that tumors with IDH mutations were more sensitive to cisplatin, dasatinib, and erlotinib than those without IDH mutations (IDH-wt, **Figure 6C**).

Moreover, we found that LGG was most sensitive to cisplatin and erlotinib when high expression of PTPRN or RIM-BP2 was combined with the IDH mutation. Therefore, cisplatin and erlotinib are preferred chemotherapies in LGG with IDH mutations and high PTPRN and RIM-BP2 expression.

### Tumor Mutational Burden of PTPRN and RIM-BP2

**Figure 7:**
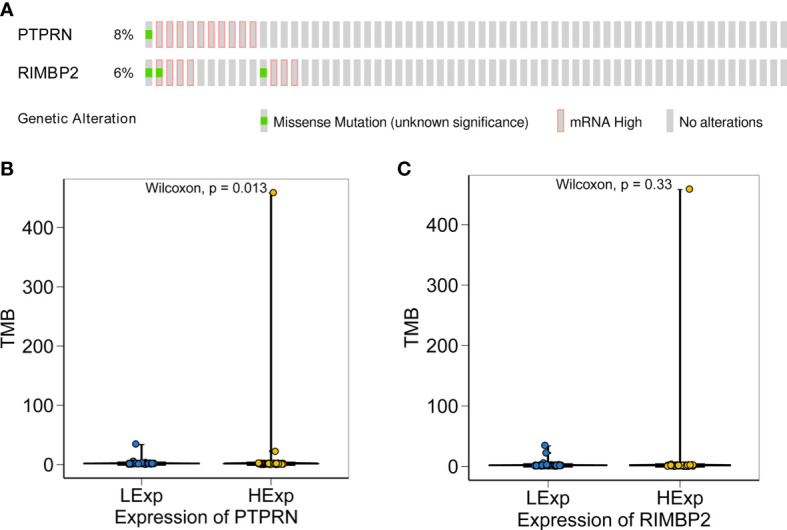
Relationship between the expression of PTPRN and RIM-BP2 and TMB score. **(A)** PTPRN and RIM-BP2 mutations in tumors. **(B, C)** The TMB score of the high PTPRN expression group was significantly higher than that of the low expression group (p=0.013). TMB score was not significantly related to the expression of RIM-BP2.

After demonstrating the effect of PTPRN and RIM-BP2 expression on the response to RT and chemotherapy, we identified the PTPRN and RIM-BP2 mutations in tumors. All GBM data sets were derived from the TCGA-Pancancer database, and TMB of PTPRN and RIM-BP2 was estimated using CIBERSORT (https://cibersort.stanford.edu/). The results showed that PTPRN and RIM-BP2 coding mutations existed in a total of 15 patients with GBM (10%). The mutation frequencies were 8% and 6% for PTPRN and RIM-BP2, respectively, in 15 patients (**Figure 7A**). Subsequently, we analyzed the relationship between PTPRN or RIM-BP2 expression and TMB score. The results showed that the TMB score in patients with GBM and low PTPRN expression was significantly higher than that of patients with high PTPRN expression (**Figure 7B**). The difference in the RIM-BP2 expression group was not statistically significant (**Figure 7C**).

### Correlation Between Immune Cell Subtypes and of the Expression of PTPRN and RIM-BP2

The immune microenvironment has been shown to play a critical role in tumor biology. Recently, numerous promising preclinical and clinical immunotherapeutic treatments and gene therapy have been achieved for GBM. However, the role of immunotherapy in gliomas needs to be further clarified (22, 23). Hence, the molecular profiles within the tumor microenvironment may be valuable predictive biomarkers.

**Figure 8:**
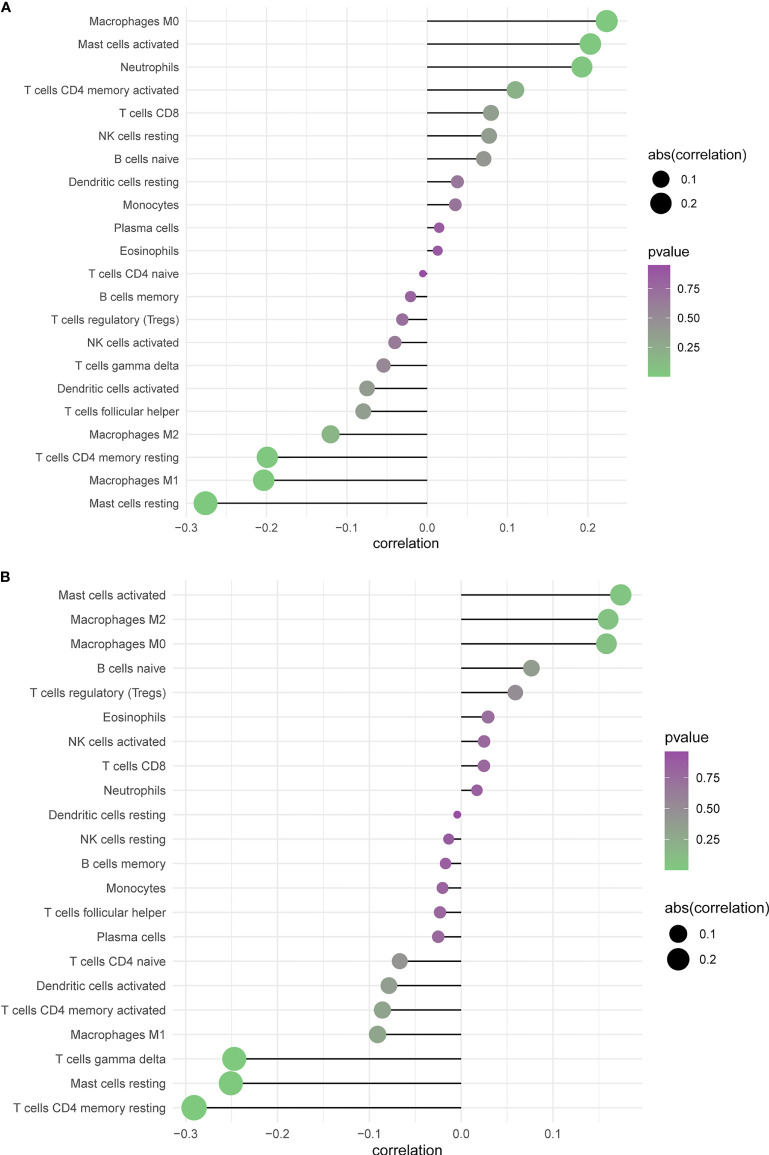
Profiling Tumor-Infiltrating Immune Cells with CIBERSORT. **(A, B)** Summarizing immune cell subset proportions in GBM against the expression of PTPRN and RIM-BP2 status and CIBERSORT p-value.

The CIBERSORT algorithm acquired the relative proportions of 22 immune cell subsets in GBM. The correlations between the proportions of the 22 immune cell subtypes and PTPRN and RIM-BP2 expression are shown in **Figure 8A**. There was a large degree of tumor immune cell infiltration, including M0 macrophages, M2 macrophages, activated mast cells, neutrophils, and resting memory CD4 T cells, in patients with GBM and high PTPRN expression. At the same time, there was also massive tumor immune cell infiltration, such as M2 macrophages, M0 macrophages, resting memory CD4 T cells, and gamma delta T cells, in patients with GBM and high expression of RIM-BP2 (**Figure 8B**). Combining the prognosis analysis, RT and chemotherapy sensitivity analysis, and tumor infiltrated immune cell subsets analysis, we concluded that patients with higher expression of PTPRN and RIM-BP2 were resistant to RT and chemotherapy, potentially due to poor tumor microenvironment; therefore, their prognosis was very poor.

## Discussion

By normalizing and analyzing the GSE12657, GSE90886, and GSE90598 datasets, we identified 141 significantly overlapping DEGs in GBM. By overlapping biomarkers derived from the LASSO algorithm and the Boruta algorithm, we obtained five GBM biomarkers. According to Kaplan-Meier survival curve analysis, high PTPRN or RIM-BP2 expression was shown to predict poor OS.

RIM-binding protein 2 (RIM-BP2), a multidomain cytomatrix protein, is present at the inner hair cell active zones (24). RIM-BP2 has diversified functions in neurotransmitter release at different central murine synapses and thus contributes to synaptic diversity (25). However, little work has been done to elucidate the expression and role of RIM-BP2 in cancer. Our study found for the first time that RIM-BP2 was significantly downregulated in GBM and that high RIM-BP2 expression was strongly associated with poor prognosis in patients with GBM. These results suggested that in the molecular pathogenesis, progression, and prognosis of GBM, RIM-BP2 may play an important role. PTPRN is a gene that encodes the protein tyrosine phosphatase receptor type N, a 105.8-kDa protein from the tyrosine phosphatase (PTP) family responsible for signaling related to cancer initiation and progression (26, 27). PTPRN is abnormally expressed in many tumors, including small cell lung cancer (SCLC), breast cancer, and liver cancer, and affects tumor progression. We found that high PTPRN expression is strongly associated with a poor prognosis in patients with GBM, which was consistent with previous findings (28–30).

Studies to identify and validate protein targets to improve the therapeutic options are underway. We further analyzed whether the current treatment options for GBM, including RT and chemotherapy, are beneficial even when the PTPRN or RIM-BP2 expression in glioma is abnormal.

The RT regimen of 60 Gy for six weeks has long been the standard adjuvant approach for GBM. It remains the primary treatment modality for unresectable GBM and prolongs survival (31, 32). The results showed that the expression of PTPRN was related to the sensitivity of RT. The GSVA database showed that the differential expression of PTPRN is involved in the DNA repair pathway. Moreover, our results also showed that the activation of DNA repair pathways is correlated with low PTPRN expression (p=1.989e-04). An enhanced cellular DNA repair system is recognized as a major cause of RT failure and, accordingly. GBM is often resistant to RT due to enhanced DNA repair activity (33). Our results implied that low PTPRN expression in GBM is associated with the activation of DNA repair systems as defense mechanisms underlying radioadaptive protection.

TMZ is part of the standard chemotherapeutic regimen for GBM (34). As an alternative, targeted therapies can limit harmful toxicity and more effectively block tumor proliferation. The use of existing clinical data to model the tumor dynamic response to antitumor treatments is a promising approach toward improving treatment efficacy and accelerating the development of antitumor drugs. To identify targets for GBM treatment, Andrea Shergalis, using data from TCGA, discovered 20 genes, including PTPRN, that correlated with poor survival outcomes, which was consistent with our findings (35). However, the author did not further discuss the possible chemotherapy drugs that might target these genes. In our study, the prediction model of the GDSC was used to evaluate chemotherapy drugs according to the IC50 value. There were significant differences for several drugs according to the *PTPRN* and *RIM-BP2* expression. PTPRN in GBM is more susceptible to AKT inhibitor VIII, cisplatin, erlotinib, gefitinib, and gemcitabine. RIM-BP2 may be more sensitive to AKT inhibitor VIII, cisplatin, dasatinib, erlotinib, gefitinib, and gemcitabine. To explore the chemotherapy drugs targeting IDH mutation in LGG, we identified drugs with different estimated IC50 values for the IDH mutation compared to IDH-wt. Our results showed that the estimated IC50 values of chemotherapeutic drugs (cisplatin, dasatinib, and erlotinib) are different between the IDH-wt and IDH-mutation groups. The chemotherapy drugs cisplatin and erlotinib had significant impact in GBM, LGG, and tumor with IDH mutations. There is no standard approach for the successful treatment of recurrent brain tumors. Cisplatin and erlotinib may provide a new line of chemotherapy for gliomas.

Cisplatin has been approved for use as an antitumor drug for approximately forty years, and the antitumor efficacy of cisplatin is unquestionable (36). The proposed treatment protocol based on a combination of carboplatin and vincristine, first reported in 1993, has achieved high objective response rates of 52% and 62%, respectively, in relapsed and newly diagnosed LGG patients (37). Although cisplatin is used for adjuvant chemotherapy against glioma, intrinsic and acquired resistance restricts cisplatin application (38). Erlotinib, a tyrosine kinase inhibitor, has shown promising response rates in malignant gliomas. Among glioma patients, those with glioblastoma multiforme tumors who have high EGFR expression levels and low levels of phosphorylated PKB/Akt had a better response to erlotinib treatment (39). However, although this targeted compound performed well in preclinical studies, it has failed phase II clinical trials in humans (40, 41). Ultimately, several factors are responsible for drug treatment failure, including toxicity and the failure of the compounds to reach effective concentrations in the brain (40).

TMB is a promising marker of response to immune therapy (IT) that is emerging as a new predictive biomarker to select patients who may benefit from immune checkpoint inhibitor therapy (ICI) (42). High TMB can increase the number of neoantigens that recruit the adaptive immune system and thus provide a potential biomarker for response to IT. In recent years, an association between clinical benefit and high TMB was observed in some human cancers (43, 44). The TMB cutoff points associated with improved survival vary markedly between cancer types, and there may not be one universal definition of high TMB (45). Interestingly, our results showed that the TMB score of the low PTPRN expression group was significantly higher than that of the high expression group (p=0.013). M0 macrophages, M2 macrophages, activated mast cells, neutrophils, and resting memory CD4 T cells comprise a large proportion of PTPRN-related immune cell infiltrates. The difference in the RIM-BP2 expression group was not statistically significant. Whether PTPRN expression, identified in our study as a novel biomarker,is a potential predictor of GBM prognosis related to TMB needs further investigation. PTPRN-related immune cell infiltration is more likely to be a response to immunotherapy, providing us with new insights and opportunities to further investigate its association with disease course and response to therapy.

There are some limitations to our work. First, the sample size included in our analysis was small, which might lead to the omission of some potential messenger RNAs (mRNAs). Moreover, the expression of PTPRN and RIM-BP2 was only detected using bioinformatics analysis and require further experimental verification in more patients with GBM. Third, we did not validate the prognostic value of PTPRN and RIM-BP2. Furthermore, we provided some potential treatment options relating to PTPRN, including radiotherapy and chemotherapy. The molecular mechanisms of how the gene signatures and treatment selection affect the prognosis of GBM should be further elucidated.

In conclusion, we identified two novel biomarkers (PTPRN and RIM-BP2) that can potentially be used for prognosis prediction in GBM. These genes have potential clinical implications for radiotherapy and chemotherapy GBM treatment. However, the molecular mechanism and function of these genes need to be confirmed in further experiments.

## Data Availability Statement

The original contributions presented in the study are included in the article/**Supplementary Material**. Further inquiries can be directed to the corresponding authors.

## Author Contributions

SL and YH conceptualized and designed the study. SP participated in the bioinformatics analyses. XZ drafted the manuscript. RL and ZC participated in the design of the study. XX helped to revise the study. All authors contributed to the article and approved the submitted version.

## Funding

This work was supported by the Scientific research project (2019) of the health commission of Hunan (grants B2019200) and the Science and technology innovation project of Hunan(grants 2018SK52802).

## Conflict of Interest

The authors declare that the research was conducted in the absence of any commercial or financial relationships that could be construed as a potential conflict of interest.

## Publisher’s Note

All claims expressed in this article are solely those of the authors and do not necessarily represent those of their affiliated organizations, or those of the publisher, the editors and the reviewers. Any product that may be evaluated in this article, or claim that may be made by its manufacturer, is not guaranteed or endorsed by the publisher.
