# Supplementary material for: Novel Biomarker Genes for Prognosis of Survival and Treatment of Glioma
Source: Front Oncol. 2021 Dec 15;11:667884. doi: 10.3389/fonc.2021.667884 (PMC8714878; doi:10.3389/fonc.2021.667884)
Supplement: Supplementary file 1 [file DataSheet_1.doc]

**Supplementary Table1**

141 Differentially expressed genes

| Differentially expressed genes | |
| --- | --- |
| Up-regulated | F2R, IFI44, TNC, COL4A1, EZH2, COL4A2, COL6A2, LAMC1, ANGPT2, BAZ1A, VEGFA, FCGBP, TOP2A, PYGL, TPX2, CKS2, ABCC3, TIMP4, UBE2C, IGFBP2, SERPINE1, TFPI, IGF2BP3, COL3A1, PTX3, TGFBI, LYZ, SERPINA3, POSTN, LTF |
| Down-regulated | SLC8A2, PTPRN, TBR1, GABRA5, KCNAB2, ICAM5, ERC2, SPTBN2, PPFIA3, HCN2, DDN, GRIN1, SNCG, CAMK1G, RIMBP2, SYN2, STX1A, KCNJ4, GABRA2, KCNJ3, DYNC1I1, SLC17A7, GAD2, RIMS3, CACNG3, MAPK8IP2, SV2B, NELL1, NPTXR, SLC6A15, NMNAT2, BCL11A, PTPRR, PDE2A, KIAA0319, CHD5, KCNQ2, RUNDC3A, CA11, PHYHIP, RIMS2, RBP4, PPP1R16B, MYT1L, SLC12A5, RAPGEF4, PCLO, SH3GL3, PAK3, SH3GL2, EPHB6, NPTX1, ATRNL1, SNAP91, AMPH, FRRS1L, CAMK2A, DLG2, PSD, CABP1, ERBB4, GRIN2A, NELL2, ANO3, PRKCZ, SYT1, BZRAP1, RCAN2, NEFM, CCK, INA, CDS1, CHGA, CALB2, RTN1, SPOCK3, STMN2, CDH18, RALYL, SYP, PDYN, GRM3, GABRG2, NRGN, TPPP, LMO3, KIAA1107, CRYM, ANK3, SERPINI1, RGS7, NEFL, GRM5, SST, PRKCB, CLDN10, FXYD1, KALRN, SULT4A1, HTR2A, RGS4, VSNL1, CHGB, CUX2, SNAP25, KCNK1, MAL, GABRB1, MAG, WIF1, SNCB |

**Supplementary Figure 1** GO and KEGG pathway enrichment analysis performed with up-regulated genes


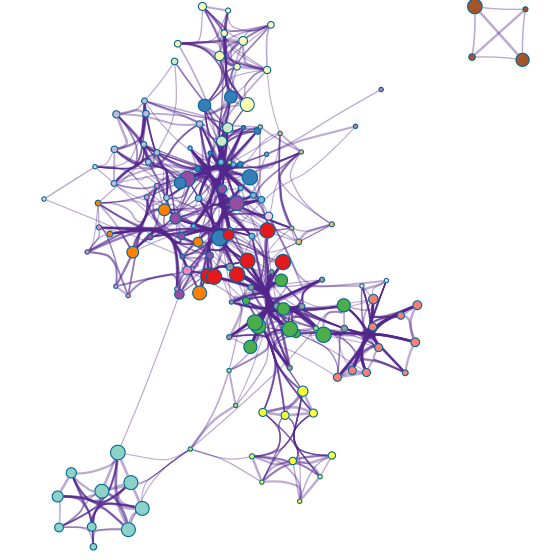

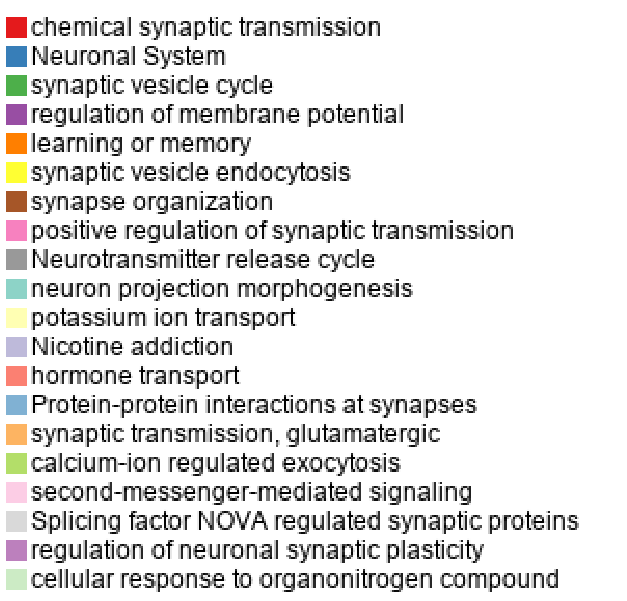

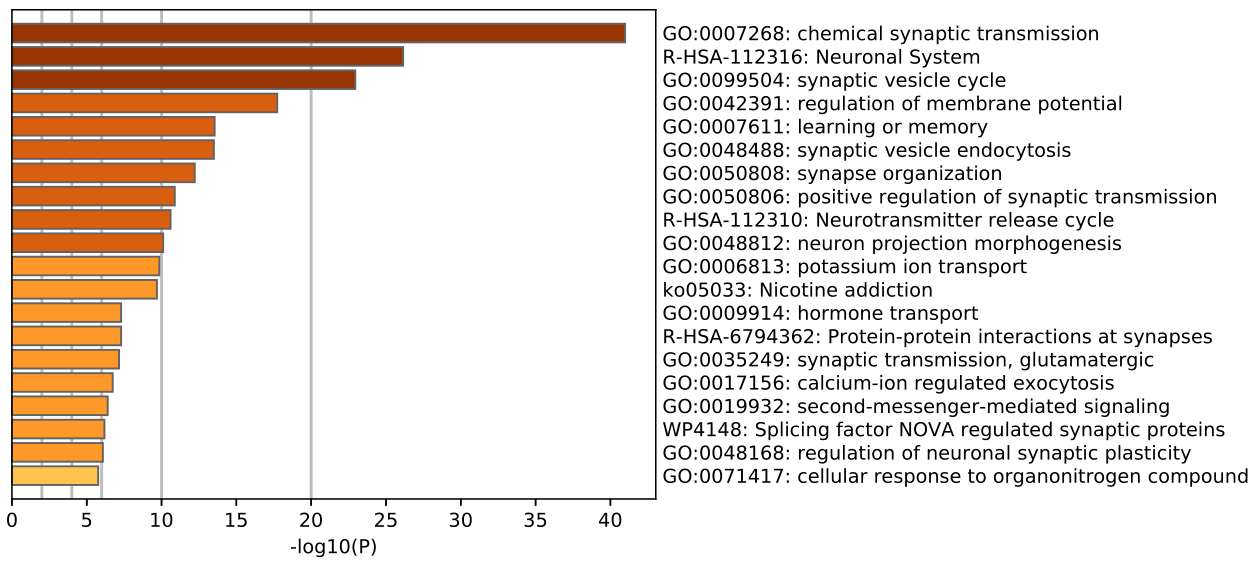

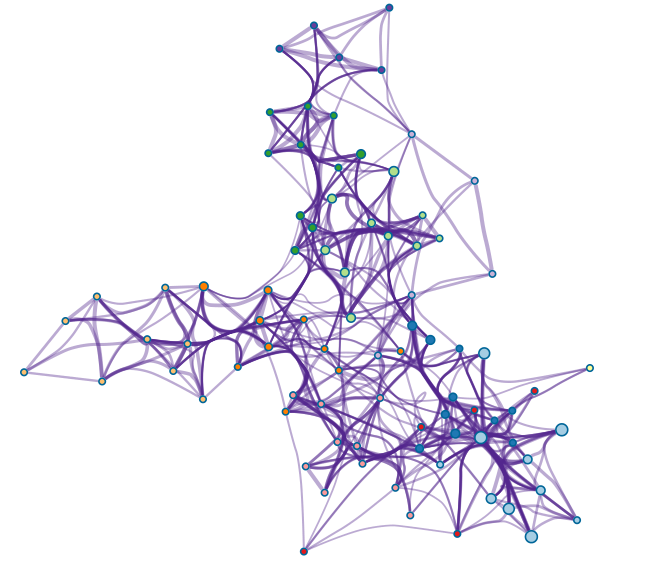

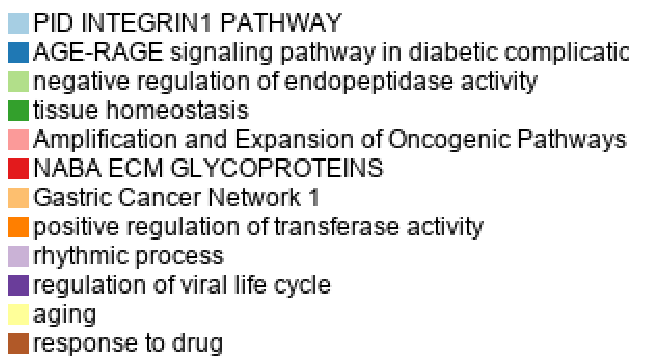


**Supplementary Figure 2** GO and KEGG pathway enrichment analysis performed with down-regulated genes
